# Structural Basis of Prolyl Hydroxylase Domain Inhibition by Molidustat

**DOI:** 10.1002/cmdc.202100133

**Published:** 2021-04-09

**Authors:** William D. Figg, Michael A. McDonough, Rasheduzzaman Chowdhury, Yu Nakashima, Zhihong Zhang, James P. Holt‐Martyn, Alen Krajnc, Christopher J. Schofield

**Affiliations:** ^1^ Department of Chemistry University of Oxford 12 Mansfield Road Oxford OX1 3TA UK; ^2^ Cardiovascular Research Institute University of California, San Francisco 555 Mission Bay Blvd. San Francisco CA 94158 USA; ^3^ Institute of Natural Medicine University of Toyama 2630 Sugitani Toyama 930–0194 Japan

**Keywords:** oxygenases, anaemia, hypoxia-inducible factor-alpha (HIF), Molidustat, enzyme inhibition

## Abstract

Human prolyl‐hydroxylases (PHDs) are hypoxia‐sensing 2‐oxoglutarate (2OG) oxygenases, catalysis by which suppresses the transcription of hypoxia‐inducible factor target genes. PHD inhibition enables the treatment of anaemia/ischaemia‐related disease. The PHD inhibitor Molidustat is approved for the treatment of renal anaemia; it differs from other approved/late‐stage PHD inhibitors in lacking a glycinamide side chain. The first reported crystal structures of Molidustat and IOX4 (a brain‐penetrating derivative) complexed with PHD2 reveal how their contiguous triazole, pyrazolone and pyrimidine/pyridine rings bind at the active site. The inhibitors bind to the active‐site metal in a bidentate manner through their pyrazolone and pyrimidine nitrogens, with the triazole π‐π‐stacking with Tyr303 in the 2OG binding pocket. Comparison of the new structures with other PHD inhibitor complexes reveals differences in the conformations of Tyr303, Tyr310, and a mobile loop linking β2–β3, which are involved in dynamic substrate binding/product release.

## Graphical abstract

**Ring binder**: Co‐crystal structures of human PHD2 with Molidustat and a related inhibitor provide insight into their mode of inhibition. The pyrazolone‐pyrimidine rings of Molidustat chelate the active‐site metal ion, and its triazole ring makes a π‐π‐stacking interaction with Tyr303. The results reveal altered conformations of PHD2 residues, including Tyr303 and Tyr310 on binding of Fe‐chelating PHD inhibitors.

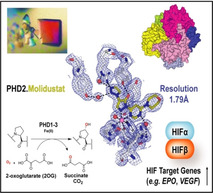

Human prolyl‐hydroxylases 1‐3 (PHDs) are hypoxia‐sensing 2‐oxoglutarate (2OG) and Fe^II^‐dependent oxygenases that regulate levels of the hypoxia‐inducible transcription factor α‐subunits (HIFα). The PHDs catalyse *trans‐*4‐prolyl hydroxylation of proline‐residues of the HIFα N‐ and C‐terminal oxygen degradation domains (NODD and CODD), reactions coupled to conversion of dioxygen and 2OG to succinate and carbon dioxide (Figure 1A, B).^[1]^ Hydroxyproline formation promotes HIFα degradation through stabilisation of its interaction with the von Hippel‐Lindau (pVHL) protein‐E3 ligase complex resulting in HIFα ubiquitination and proteasomal proteolysis.^[2]^ There are three human PHDs (PHD1‐3) and three HIFα isoforms (HIF1‐3α). In hypoxia, PHD activity reduces, HIFα levels rise, HIFα translocates into the nucleus, and dimerises with HIFβ to form transcriptionally active α,β‐HIF that binds to hypoxic response elements (HRE) of target genes (Figure 1A and D). Interactions with other proteins involved in transcriptional regulation, including coactivator proteins leads to context‐dependent upregulation of multiple HIF target genes, including erythropoietin (*EPO*) and vascular endothelial growth factor (*VEGF*).^[3]^

**Figure 1:**
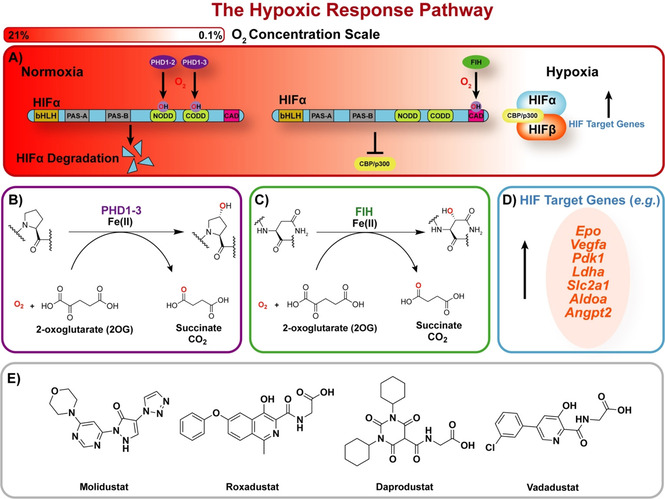
Overview of the HIF‐mediated hypoxic response. A) In normoxia, the PHDs hydroxylate HIFα‐oxygen‐dependent degradation domain (ODD) sequences leading to efficient HIFα degradation *via* the E3‐ligase ubiquitination pathway. FIH is more active than the PHDs in moderate hypoxia and hydroxylates an asparagine‐residue in the HIFα‐C‐terminal transactivation domain preventing the CBP/p300 transcriptional enhancers from binding to HIF. In hypoxia, the PHDs/FIH are less active enabling active HIFα/β formation and upregulation of HIF target genes. B) PHD1‐3 use Fe^II^, 2OG, and O_2_ to catalyse 4‐hydroxyproline‐residue formation. C) FIH similarly catalyses conversion of an asparagine to a 3*‐*hydroxyasparagine residue. D) Examples of HIF target genes. E) Structures of Molidustat and selected PHD inhibitors approved for clinical use/in late‐stage development.

Factor inhibiting HIF (FIH), a 2OG oxygenase from a different structural subfamily to the PHDs, catalyses hydroxylation of an asparagine‐residue in the HIFα C‐terminal transactivation domain (CAD), a modification hindering HIF binding to the transcriptional coactivators CBP/p300 (Figure 1A and C).[^4^, ^5^] FIH catalysis is likely involved in regulating the set of HIF target genes upregulated in a given context.^[6]^ For some applications, it might be advantageous to have simultaneous inhibition of both the PHDs and FIH. In other cases, selective PHD inhibition may be desirable.^[6]^

PHD inhibition is a promising treatment of anaemia in chronic kidney disease by promotion of EPO‐mediated erythropoiesis and has potential to treat other ischaemia‐related disorders.^[7]^ Pioneering PHD inhibition studies employed a strategy mimicking 2OG binding, exemplified by *N*‐oxalylglycine (NOG) and its prodrug, dimethyloxalylglycine.^[8]^ Most first‐generation PHD inhibitors bind to the active site Fe^II^ in a bidentate mode, analogously to the 2OG oxalyl group, and have a glycinamide side chain occupying the pocket filled by the 2OG methylenes and C5 carboxylate.[^9^, ^10^] It is proposed that clinically applied PHD inhibitors are desirably selective for the PHDs and/or FIH with low activity against the other ∼70 human 2OG oxygenases.^[11]^ Achieving selective inhibition of the PHDs with 2OG analogues is challenging; further, the glycinamide side chain might be amenable to metabolism, for example by glucuronidation.^[12]^

The development of Molidustat (BAY 85‐3943, which is approved in Japan for treatment of renal anaemia), which contains contiguous triazole, pyrazolone, pyrimidine and morpholine rings, is of interest because it lacks the glycinamide side chain (a 2OG mimic), present in other PHD inhibitors in clinical trials, such as Vadadustat (AKB‐6548), Roxadustat (FG‐4592), and Daprodustat (GSK1278863; Figure 1E and Figure S1 in the Supporting Information).[^13^, ^14^, ^15^] In the related compound IOX4 (Figure S1), the morpholine ring of Molidustat is replaced with a *tert*‐butyl ester to enable brain penetration.^[9]^ Molidustat and IOX4 are potent inhibitors with reported half‐maximal inhibitory concentration (IC_50_) values of 7 and 3 nM, respectively, for isolated PHD2.

A crystal structure of FIH complexed with Molidustat is reported (PDB ID: 5OP8), though by contrast with its potent PHD2 inhibition (IC_50_ 7 nM), Molidustat only weakly inhibits FIH (IC_50_ 66 μΜ).^[10]^ Thus, the binding mode of Molidustat to FIH is not necessarily representative of that with the PHDs. A low‐resolution (3.3 Å) structure of PHD2 complexed with IOX4‐A, IOX4 lacking its *tert*‐butyl ester group, (IC_50_ 4.8 nM, PDB ID: 5A3U) showing bidentate binding of the IOX4 pyrazolone‐pyridine rings to the active site metal ion is reported (Figure S1).^[9]^ However, no structures of human PHD1‐3 in complex with Molidustat or IOX4 are available.^[10]^ In addition to the Fe^II^ chelation/2OG competition typically observed, PHD inhibition may also involve conformational changes, likely relating to those involved in HIFα substrate binding.^[10]^ The lack of structural information for PHD2 in complex with Molidustat might thus reflect conformational heterogeneity, for example, involving the β2–β3 “finger loop”, which folds to enclose the active site and the loop residues interact with HIFα substrates.[^10^, ^16^] However, such conformational heterogeneity has not prevented solution of PHD2 structures with potent inhibitors such as FG‐2216 (*P*6_3_ space group, Figures S1 and 4E) or Vadadustat (*P*6_3_ space group_,_ Figures S1 and 2C).[^10^, ^17^] Here we report the identification and use of a previously unreported crystal form (*P*2_1_ space group) that enables determination of PHD2 structures with Molidustat (PDB ID: 6ZBO, 1.79 Å) and IOX4 (PDB ID: 6BZN, 2.01 Å). The work reveals important interactions and identifies the importance of dynamic interactions during inhibition, including π‐π‐stacking interactions with Tyr303, electrostatic interactions with active site waters‐Arg383‐Tyr329, and the β2–β3 loop.

A new crystallisation condition was obtained through a broad screen and resulted in the formation of rhombohedral PHD2_181‐407_ crystals when using potassium thiocyanate and poly(ethylene glycol) 3350 (Table S1). PHD2_181‐407_‐Molidustat (PDB ID: 6ZBO) and IOX4 (PDB ID: 6ZBN) complexes were obtained through co‐crystallisation and diffracted to 1.79 Å and 2.01 Å resolution, respectively.

The crystal form diffracted in the *P*2_1_ space group with six PHD2_181‐407_ molecules in the asymmetric unit (ASU, chains A–F, Figure S2); PHD2 is principally monomeric in solution.[^18^, ^19^] The Glu407 side chain in chains A, C, and E is positioned to form a salt‐bridge interaction with the Arg396 side chain in chains D, B, and F, reflecting interactions between helix α4 of adjacent protomers (Figures 2A and S2). The βII strands of the double‐stranded β‐helix (DSBH) 2OG oxygenase core fold of PHD2‐Molidustat chains A, B, C, D, E, and F interact with the βV strands of chains B, F, D, E, C, and A, respectively, through antiparallel interactions (Figure S2). Comparison of the deviation (RMSD) values (Table S4) indicates small variations between chains A–F (backbone RMSDs 0.17–0.35 Å). Chain A is used in subsequent descriptions and analysis of PHD2_181‐407_ Molidustat and IOX4 complexes.

**Figure 2:**
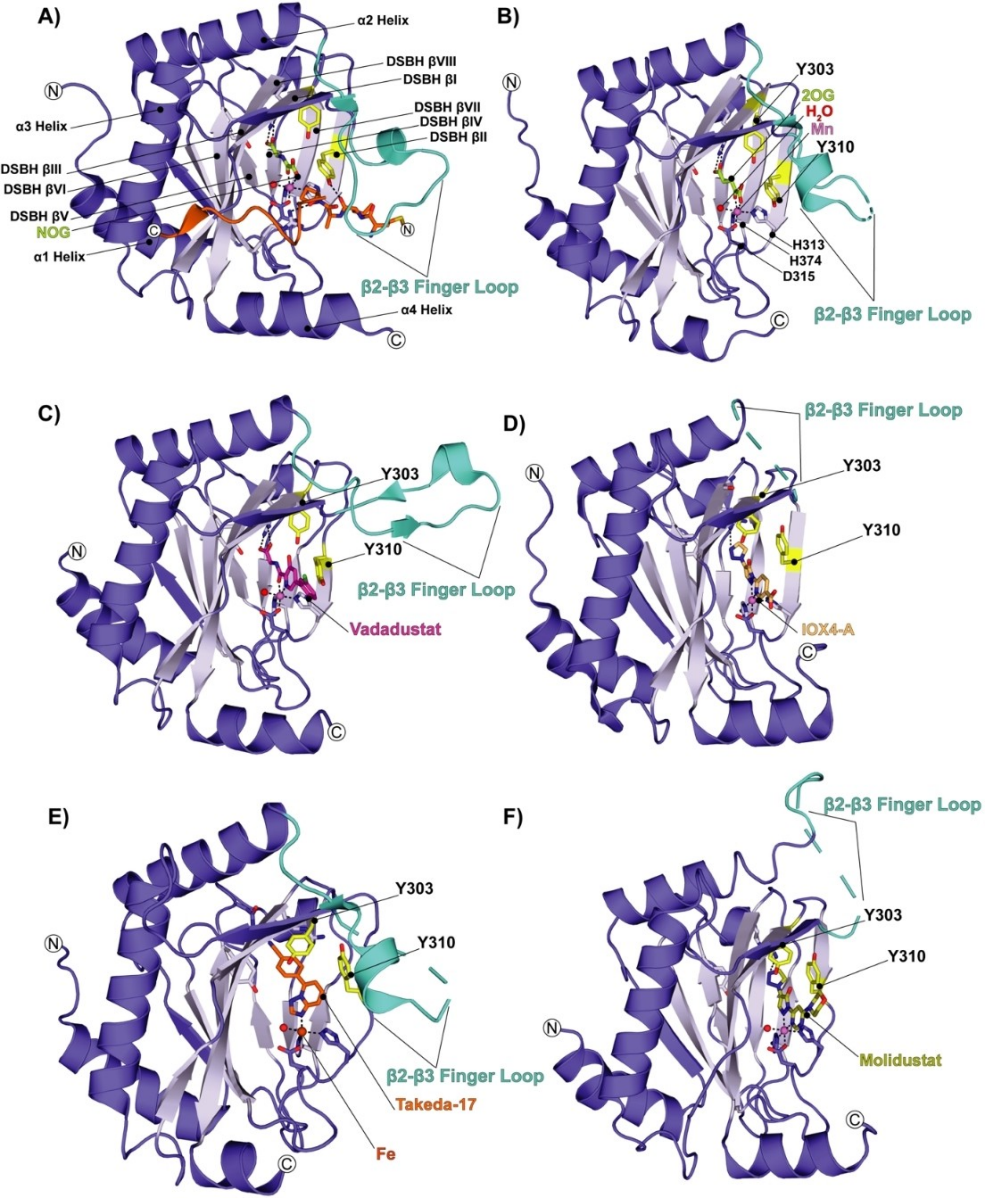
Conformational changes of PHD2 and the β2–β3 finger loop between substrate, co‐factor, and inhibitor structures. Key residues are in yellow, and the β2–β3 finger loop is in cyan. Views of: A) PHD2‐NOG (lemon)‐HIF1α‐CODD (orange) highlighting the DSBH fold; (PDB ID: 3HQR). B) PHD2‐2OG (lemon; PDB ID: 3OUH). C) PHD2‐Vadadustat (magenta) complex (PDB ID: 5OX6). D) PHD2‐IOX4‐A (light orange); note the disordered β2–β3 finger loop (PDB ID: 5A3U). E) PHD2‐Takeda‐17 (orange); note the disordered β2–β3 finger loop (PDB ID: 5V18). F) PHD2‐Molidustat (olive); note the “open” position of the β2–β3 loop (PDB ID: 6ZBO, chain A).

In the *P*2_1_ crystal form, the β2–β3 finger loop residues (234–253), which are involved in substrate binding by the PHDs, are disordered between residues 231–253 (Figure 2F).^[18]^ Partial disorder of the β2–β3 finger loop residues is also observed in the PHD2‐2OG complex (residues 244–249, *P*4_1_ space group), the PHD2‐IOX4‐A complex (residues 232–252, *P*3_2_12 space group), and the PHD2‐Takeda‐17 complex structure; Takeda‐17 inhibits *via* monodentate Fe^II^ coordination of its triazolopyridine ring (residues 244–254, *P*4_1_ space group; Figure 2B, D and E). By contrast, structures of PHD2_181‐426_‐HIF1α_558‐574_ (PDB ID: 3HQR, Figure 2A, *P*2_1_2_1_2_1_ space group) and PHD2_181‐426_‐Vadadustat (PDB ID: 5OX6, *P*6_3_ space group, Figure 2C) manifest electron density for all the residues of the β2–β3 finger loop.[^9^, ^10^, ^18^, ^20^]

As in other reported PHD2 structures, in the Molidustat and IOX4 complexes, the active‐site metal is coordinated by a conserved triad of residues: His313, Asp315, and His374 (Figures 2A, B and 3). During catalysis, 2OG coordinates the metal in a bidentate manner through a C1 carboxylate oxygen (*trans* to His374) and the C3 ketone oxygen (*trans* to Asp315); the 2OG C5 carboxylate forms a salt bridge with Arg383 (Figure 2B). Molidustat and IOX4, bind in a similar mode to the metal ion, that is, through bidentate chelation of their pyrimidine/pyridine N1 (2.15 Å/2.14 Å, *trans* to His374 NE2) and pyrazolone (2.27 Å/2.24 Å, *trans* to Asp315 OD1) nitrogens (Figure 3D); the distance values reported here and subsequently are as observed in chain A of the PHD2‐Molidustat and PHD2‐IOX4 complex structures, respectively.

**Figure 3:**
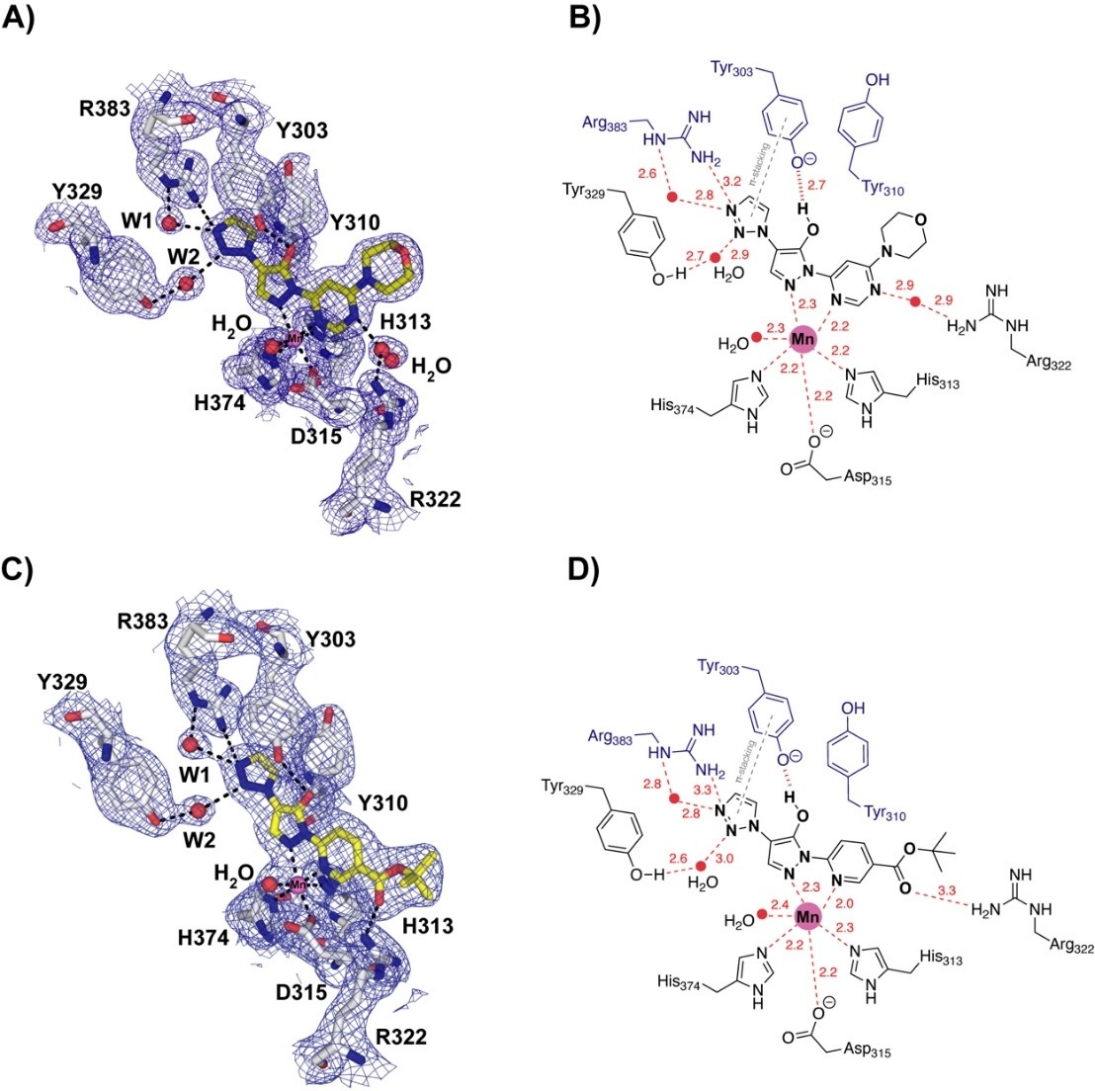
Electron‐density maps for the PHD2–Molidustat and IOX4 complexes and key active site interactions. A) 2*mF*_O_‐*F*_C_ electron density map of the PHD2‐Molidustat (olive) complex with key residues shown (chain A). B) 2D PHD active site interactions with Molidustat. C) 2*mF*_O_‐*F*_C_ electron density map of the PHD2‐IOX4 (yellow) complex with key residues shown (chain A). D) 2D PHD active site interactions with IOX4. A)–D) Note the pyrazolone ring is likely to be in its enol form; W1 and W2 are two active‐site water molecules that interact with the triazole side chain, Arg383, and Tyr329.

Consistent with the established role of triazoles as carboxylate bioisosteres, those of Molidustat and IOX4 are located in a similar manner in the predominantly hydrophobic (except for Arg383 and Tyr329) 2OG C5 carboxylate binding pocket of PHD2 (Figures 2B, 3 and 4B). The binding mode of the triazoles differs from that of 2OG and close analogues such as the glycinamide containing inhibitors (e. g., FG‐2216 and Vadadustat, Figure 3 and 4). In the case of 2OG, the C5 carboxylate forms electrostatic interactions with the two terminal guanadino NH1‐2 groups of Arg383 (2.85 and 2.94 Å, PDB ID: 3OUJ) and to make a hydrogen (H) bonding interaction with the hydroxyl side chain of Tyr329 (2.64 Å).[^10^, ^17^, ^20^] The analogous carboxylate of the glycinamide side chains of certain bicyclic and monocyclic PHD inhibitors (e. g., FG‐2216 and Vadadustat) interacts with Arg383 (2.59–2.79 Å and 2.67–2.88 Å, respectively) and Tyr329 (2.62 and 2.56 Å, respectively) (PDB ID: 4BQX and 5OX6).^[10]^ By contrast, the triazoles of Molidustat/IOX4 make a direct interaction with only one of the terminal guanadino NH(2) groups of Arg383 (3.27 Å/3.25 Å), with another being made indirectly via a water molecule (W1, Figure 3). The triazoles do not make a H bond with Tyr‐303, instead π‐π stacking with this residue, which makes a H bond with the enolic form of the pyrazolone ring of Molidustat/IOX4. Finally, the triazole N2 makes a H bond with the phenolic OH of Tyr329 via a second water (W2) (Figure 3). In the PHD2‐Molidustat/IOX4 structure complexes, W1 interacts with both the triazole N1 (2.79 Å/2.83 Å, Figure 3A, B) and ϵ‐NH of Arg383 (2.57 Å/2.77 Å). W2 interacts with the triazole N2 (2.92 Å/2.95 Å, Figure 3A, B) and the OH of Tyr329 (2.7 Å/2.6 Å, Figure 3).^[21]^

**Figure 4:**
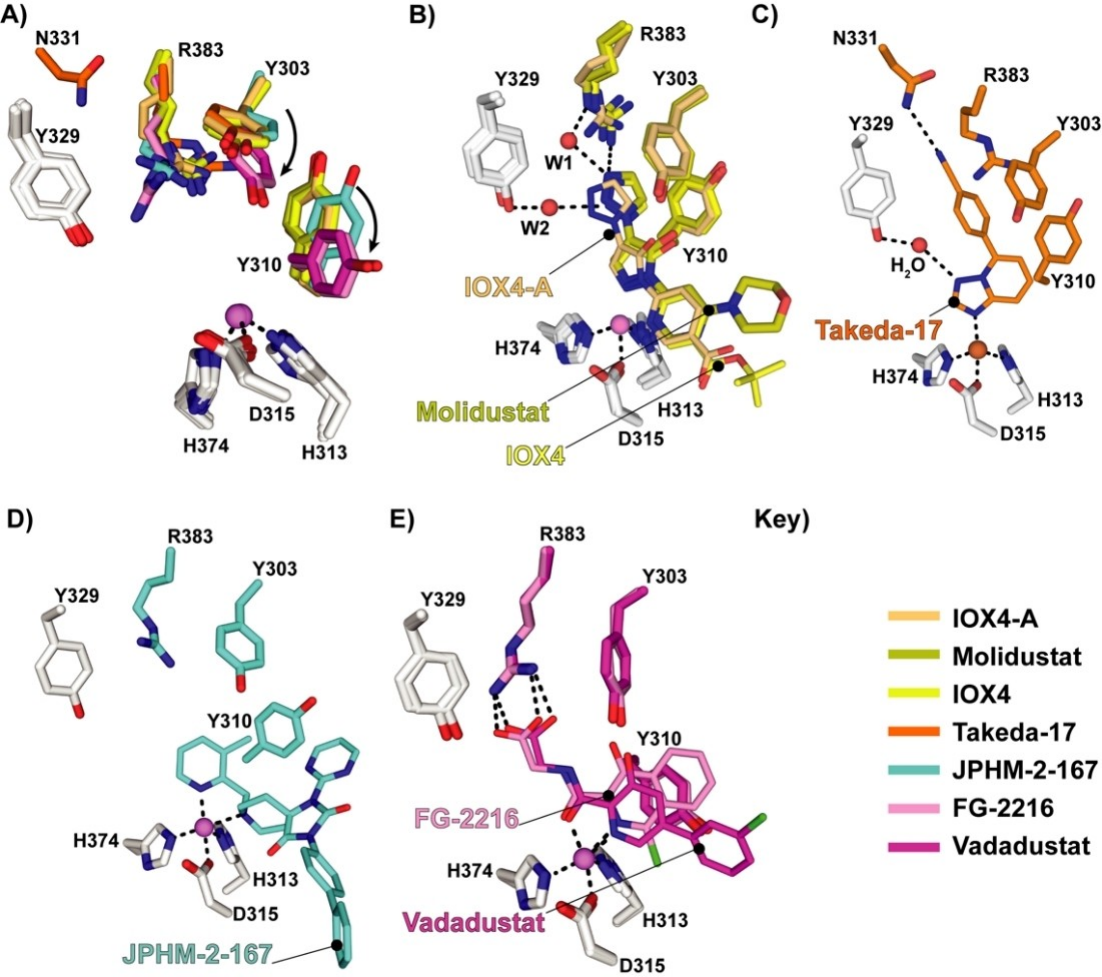
Comparison of PHD2–inhibitor complex structures (Molidustat, IOX4, Vadadustat, FG‐2216, Takeda‐17, and IOX4‐A) comparing the conformations of Tyr303, Tyr310, and Arg383. A) Superposition of inhibitor complexes showing Tyr303, Tyr310, and Arg383: FG2216 (PDB ID: 4BQX, pink), IOX4‐A (PDB ID: 5A3U, light orange), Vadadustat (PDB ID: 5OX6, magenta), Takeda‐17 (PDB ID: 5V18, orange), JPHM‐2‐167 (PDB ID: 6QGV, cyan), IOX4 (PDB ID: 6ZBN, yellow, chain A monomer), and Molidustat (PDB ID: 6ZBO, olive, chain A monomer). Note the different conformations of Tyr303, Tyr310, and Arg383. B) Views of: PHD2‐Molidustat (PDB ID: 6ZBO, olive, chain A monomer), IOX4 (PDB ID: 6ZBN, yellow, chain A monomer), and IOX4‐A (PDB ID: 5A3U, light orange) complexes. C) PHD2‐Takeda‐17 (PDB ID: 5V18, orange) complex. D) PHD2‐JPHM‐2‐167 (PDB ID: 6QGV, cyan) complex. E) PHD2‐Vadadustat (PDB ID: 6QGV, cyan) and FG‐2216 (PDB ID: 4BQX, pink) complex.

The structures imply that both Molidustat and IOX4 probably bind at the active site with their pyrazolone ring (at least) predominantly in its enol form, as supported by the likely presence of a H bond between the enol and the phenol of Tyr303 (2.63 Å/2.71 Å) and metal ligation *via* the imine (N2) pyrazolone nitrogen (Figure 3).^[22]^ The Molidustat and IOX4 morpholine and *tert*‐butyl ester carboxylate side chains, respectively, extend out of the active site.

During PHD catalysis, the guanidine NH1 of Arg322 H bonds with the backbone carbonyl of Pro564 of its HIF1α‐CODD substrate (2.65 Å) and likely makes an analogous interaction with other ODD substrates (PDB ID: 3HQR).^[18]^ Both Molidustat and IOX4 compete with 2OG, but incompletely displace the HIFα substrates.[^9^, ^10^] The interaction between the *tert*‐butyl ester carbonyl of IOX4 and Arg322 may in part reflect the observation that IOX4 displaces HIF1α‐NODD more efficiently than Molidustat, though the details of how this occurs are unclear and may involve correlated motions.[^9^, ^10^]

Potential crystallisation conditions influence PHD protein dynamics (Table S3) and so may promote or hinder nucleation of particular conformations.^[23]^ Thus, at least to some extent, the precise nature of the available PHD structures likely reflects the varied conditions used for crystallisation (Table S3), which has been done using a range of ligands including both tight and weak binding inhibitors as well as substrate noncompetitive cyclic peptides binding away from the active site.^[23]^ Nonetheless, it is of interest to compare our Molidustat/IOX4 structures with those of other triazole containing (IOX4‐A, and Takeda‐17) and non‐triazole‐containing inhibitors (e. g., Vadadustat and FG‐2216) with respect to interactions with Tyr303, Tyr310, Tyr329, and Arg383 (Figures 4 and S4).[^9^, ^24^] Tyr303, Tyr310, and Arg383 are conserved in the animal PHDs and bacterial orthologues.[^16^, ^24^] Whereas the conformation of Tyr329 appears relatively stable, those of Tyr310, Tyr329, and Arg383 vary substantially, with Tyr303 and Tyr310 being observed in different conformations in structures of both PHD1 and PHD2 with various small‐molecule ligands (Figure 4A).[^9^, ^24^, ^25^] The Molidustat/IOX4 triazoles are positioned to π‐π stack with Tyr303 (3.55 Å/3.63 Å) and are perpendicular relative to Tyr310 (3.49 Å/3.51 Å); this interaction is not observed with the PHD2‐Vadadustat and FG‐2216 complexes (Figures 4 and S4).[^10^, ^16^, ^17^]

The results demonstrate that Molidustat and related compounds inhibit PHD2, and by implication other PHDs, *via* binding of the enol form of their pyrazolone ring in a manner that competes with 2OG and where the triazole binds in the pocket that accommodates the 2OG C5 carboxylate and methylenes during catalysis (Figure 3). However, the structures reveal that the triazole does not simply replace the 2OG carboxylate, as evidenced by the presence of additional water molecules in the 2OG binding pocket and changes (relative to structures with substrates and other types of PHD inhibitor) in the conformations of residues involved in 2OG binding, including Tyr303, Tyr310, and Arg383 (Figures 3 and 4).[^9^, ^10^]

Together with other residues, including some in its C‐terminal region, the β2–β3 finger loop (PHD2 residues 234–253) residues are involved in a coordinated induced fit process during HIFα substrate binding by PHD2, as evidenced by both crystallographic and solution NMR studies.[^19^, ^26^] The different conformations of Tyr303, Tyr310, and, to a lesser extent, Arg383 in the various structures with ligands in the 2OG binding pocket (Figure 4), imply a role for active site residues in the induced fit upon 2OG/2OG competing inhibitor binding. Studies of PHD2 variants Y303A, Y310F, and Y329F have previously shown the importance of these residues in binding monocyclic Fe^II^‐chelating PHD inhibitors, with reduced binding observed in the Y303A and Y310F variants.^[27]^ Although the details remain to be resolved, these changes also likely contribute to the ordered sequential mechanism of PHD catalysis by pre‐organising the active site region for HIFα‐ODD binding, since ODD binding is promoted by prior binding of 2OG.[^18^, ^19^, ^28^] It is notable that the extent of disorder/conformation of the dynamic β2–β3 loop, which plays a key role in productive substrate binding varies in the 2OG competing inhibitor complexes (Figure 2).[^9^, ^10^, ^19^, ^20^, ^26^] It seems likely that dynamics of the β2–β3 finger loop, and potentially other regions that are involved in the induced fit during substrate binding, are linked to changes in the 2OG binding pocket region *via* correlated motions; such motions may underlie differing extents of HIFα‐ODD substrate displacement by different types of inhibitors. Molidustat and IOX4 only weakly displace HIF1α‐CODD, as observed by NMR studies.[^9^, ^10^] There is scope for exploiting the ability of Tyr303, Tyr310, and Arg383 to adopt different conformations in the design of inhibitors; indeed at least one inhibitor type (e.g. JPHM‐2‐167) appears to target a specific conformation of Tyr310 (Figure 4D).^[25]^ It is possible that differences in dynamics in these residues in different PHD isoforms might be exploited to obtain PHD isoform selective inhibitors.

By contrast with the PHDs, with FIH the 2OG C5 carboxylate interacts with the ϵ‐NH_2_ group of a lysine (Lys214_FIH_, as is characteristic of the JmjC subfamily of 2OG oxygenases) and a tyrosine (Tyr145_FIH_, PDB ID: 1MZF, Figure S3).^[29]^ In the FIH‐Molidustat complex structure (PDB ID: 5OP8), the inhibitor triazole side chain is positioned to make electrostatic and H bond interactions with Lys214_FIH_ and Tyr145_FIH_, respectively (Figure S3). Thus, despite Molidustat/IOX4 being weaker inhibitors of FIH than the PHDs, the interactions made by the triazole ring in FIH appear to more closely reflect those made by 2OG and close analogues, such as NOG.^[10]^ Thus, interactions made by Molidustat/IOX4 away from the 2OG binding pocket are important in the case of PHD inhibition. Indeed, some recently reported potent PHD inhibitors do not exploit binding in the 2OG pocket.^[24]^

The combined observations validate the general utility of the triazole ring as a 2OG C5 carboxylate bioisostere for use in 2OG oxygenase inhibition (Figure S3).[^9^, ^10^] However, the different binding modes and structural dynamics observed for Molidustat and related inhibitors of PHD2 and FIH are clear (Figures 2 and 4). Although the precise nature of the dynamics may be difficult to define, the results indicate that empirically guided substitutions on the triazole ring and or variations of it may be productive in terms of achieving potent and selective inhibitors of specific human 2OG oxygenases.

## Conflict of interest

The authors declare no conflict of interest.

## Supporting information

As a service to our authors and readers, this journal provides supporting information supplied by the authors. Such materials are peer reviewed and may be re‐organized for online delivery, but are not copy‐edited or typeset. Technical support issues arising from supporting information (other than missing files) should be addressed to the authors.

Supplementary
