# Supplementary material for: Structural Basis of Prolyl Hydroxylase Domain Inhibition by Molidustat
Source: ChemMedChem. 2021 Apr 9;16(13):2082–8. doi: 10.1002/cmdc.202100133 (PMC8359944; doi:10.1002/cmdc.202100133)
Supplement: Supplementary file 1 — Supplementary [file CMDC-16-2082-s001.pdf]

ChemMedChem

Supporting Information

Structural Basis of Prolyl Hydroxylase Domain Inhibition by Molidustat

William D. Figg Jr, Michael A. McDonough, Rasheduzzaman Chowdhury, Yu Nakashima, Zhihong Zhang, James P. Holt-Martyn, Alen Krajnc, and Christopher J. Schofield*

Supporting Information

Table of Contents

Material and Methods	3
Supplementary Tables	7
Supplementary Figures	11
References	15

Materials and Methods

All reagents, chemicals, and solvents were from Sigma-Aldrich (Merck), Apollo Scientific, Fisher Scientific, Alfa Aesar, or Melford Biolaboratories. Crystallisation screens, plates, solid precipitants, and nylon loops were from Hampton Research or Molecular Dimensions. Molidustat was synthesised by Dr James Holt-Martyn as previously described [1], and IOX4 was from Cayman Chemicals. ChemDraw (Perkin Elmer, version 18.1) was used to draw chemical structures and create 2D active site interaction figure. PyMOL (Schrödinger, version 2.1-14.7.8) was used to create crystallographic figures. Adobe Illustrator (version 25.1) was used to prepare figures.

Recombinant $PHD2_{181-407}$ Production

Recombinant $PHD2_{181-407}$ with an *N*-terminal His_6-tag (tagged-$PHD2_{181-407}$) was produced as reported using the pET-28a(+) vector.[2] The *N*- and *C*-terminally truncated PHD2 construct (residues 181-407) has previously been shown to be catalytically active.[2] In brief, the requisite vector was transformed into *Escherichia coli* BL21(DE3) cells (New England Biolabs) and grown in 2X tryptone and yeast (2TY) media. Expression was induced at 0.6-0.8 $OD_{600\ nm}$ (3-4 h) by addition of isopropyl β-D-1-thiogalactopyranoside (IPTG) at a final concentration of 0.5 mM for 4 h at 30 °C. 100 g of harvested cells (stored at -80 °C) were freeze-thawed in 400 mL of lysis buffer (20 mM Tris-HCl pH 7.5 room temperature (RT), 0.5 M NaCl, 5 mM imidazole, and 5% (v/v) glycerol) at 4 °C in the presence of a DNaseI and ethylenediaminetetraacetic acid (EDTA)-free protease inhibitor tablet (Roche). Cells were lysed by sonication (10 minutes total elapsed time, 3 seconds on/off pulse), then centrifuged (20,000 rpm or 48,384 ×g, 4 °C, Beckman Coulter-JA-25.50 rotor-Avanti-JHC centrifuge). Tagged-$PHD2_{181-407}$ was purified by Ni^{2+} affinity chromatography using a 5 mL HisTrap™ column (GE Life Sciences). The column was charged with 5 column volumes (CV) of 100 mM

$Ni(II)SO_4$ and subsequently washed with 5 CV of lysis buffer, followed by 5 CV of elution buffer (20 mM Tris-HCl pH 7.5 RT, 0.5 M NaCl, 0.5 M imidazole, and 5% (v/v) glycerol) to remove unbound Ni^{2+}, finally using 5 CV of lysis buffer. The cell lysates (250 mL) were filtered using a 0.45 µm syringe-filter (Sarstedt) and loaded onto the HisTrap™ column at (2 mL/min flow rate). The loaded column was washed with 30 CV of wash buffer (20 mM Tris-HCl pH 7.5 RT, 0.5 M NaCl, 30 mM imidazole, and 5% (v/v) glycerol). $PHD2_{181\text{-}407}$ was eluted with elution buffer using a step gradient of increasing elution buffer (16% (v/v), 34% (v/v), and 100% (v/v)) employing 5 CV for each step. Fractions (3 mL) were analysed by SDS-PAGE for purity (estimated at >75%). The tagged-$PHD2_{181\text{-}407}$ was concentrated using a concentrator (10 kDa cut off, Amicon) (4,000 rpm or 4,255 ×g, 4 °C, 5 minutes) to approximately 5-6 mL volume. 0.25 units (U) of restriction grade thrombin (Novagen, Merck) and a final concentration of 1X thrombin cleavage buffer (10X stock of 200 mM Tris-HCl pH 8.4, 1.5 M NaCl, and 25 mM $CaCl_2$, Novagen, Merck) were added to tagged-$PHD2_{181\text{-}407}$ to cleave the His_6-tag. The cleaved protein ($PHD2_{181\text{-}407}$) was loaded onto a 300 mL Superdex® 75 gel filtration column (GE Life sciences) that had been pre-equilibrated with 1 CV of 50 mM Tris-HCl pH 7.5 RT, 100 mM NaCl, 1% (v/v) glycerol. Protein was eluted at a 1 mL/min flow rate with an isocratic gradient. Fractions (4 mL) were collected and analysed for purity (estimated at >90% by SDS-PAGE). The combined samples were concentrated to approximately 50 mg/mL, and exchanged into 100 mM 2-morpholin-4-ylethanesulfonic acid (MES) pH 5.8 buffer using a PD-10 desalting column (GE Life Sciences). The sample was loaded onto a 1 mL MonoS® column (GE Life Sciences) for ion-exchange chromatography, eluting with a linear gradient to 100% of 100 mM MES pH 5.8, 1 M NaCl over a 5 mL volume. 1 mL fractions were collected; the purity of total collected fractions was estimated to be >95% by SDS-PAGE). The combined fractions were concentrated to approximately 50 mg/mL, and exchanged into 50 mM Tris-HCl pH 7.5 RT and 1% (v/v) glycerol (final crystallisation buffer)

via a PD-10 column. $PHD2_{181-407}$ was concentrated to 26 mg/mL or 1 mM $PHD2_{181-407}$, aliquoted at 65 µL, flash-frozen in liquid nitrogen, and stored at -80 °C. Prior to freezing, the protein was characterised by SDS-PAGE (>95% pure) and liquid chromatography-electrospray ionisation mass spectrometry (LC-MS) to confirm the removal of His_6-tag and correct mass of $PHD2_{181-407}$.[2]

Crystallography

$PHD2_{181-407}$ protein (1 mM) was pre-treated with Mn(II) (1.2 mM, final concentration, substituting for Fe(II), as it is catalytically inert), and the co-product succinate (2.2 mM).[3] The $PHD2_{181-407,}$ Mn(II),

and succinate mixture was then incubated for 30-60 min with Molidustat (2.2 mM) or IOX4 (2.2 mM) dissolved in dimethylsulfoxide (DMSO) using a 100 mM stock concentration of the inhibitor (Table S1). Protein-inhibitor mixtures were centrifuged with a MicroCL 21R (Thermo Fisher Scientific) at 14,000 rpm (18,800 ×g) at 4 °C for 10 mins. $PHD2_{181-407}$ crystals were grown by sitting drop vapour diffusion using 300 nL volume drops and a protein-to-precipitant solution ratio 2:1 at 298K with 0.15 M potassium thiocyanate pH 7, using 20.5-21% (w/v) poly-ethylene glycol (PEG) 3350 as the precipitant (Hampton Research, PEG/Ion screen initial hit) (Table S1). 15-20% (v/v) glycerol diluted with the precipitant was used as a cryo-protectant. 60 µm x 50 µm x 25 µm rhombohedral shaped crystals appeared after approximately 3-days of equilibration. 180 µm x 60 µm x 40 µm (PHD2-Molidustat) and 100 µm x 100 µm x 15 µm (PHD2-IOX4) crystals were mounted in nylon loops, then cryo-cooled by plunging in liquid nitrogen. Crystals were stored under liquid nitrogen, prior to mounting on a goniometer in a cryo-stream at 100K for data collection. Data were collected remotely at I04 beamline at Diamond Light Source (UK). Detailed data collection parameters, detector, and processing information are provided in Table S2. Data were autoprocessed using XIA2, DIALS,

AIMLESS, POINTLESS, and CCP4.[4] PHASER was used to solve the structures by molecular replacement (MR) using PDB: 3HQR, with the substrate and flexible loop regions removed from this structure, as the search model.[5] The initial models were fitted to the $2mF_o$-DF_c electron density contoured to 1σ using COOT.[6] The structures were then refined through iterative cycles of PHENIX-Refine and COOT until the R_{work}/R_{free} no longer decreased and converged.[7] MolProbility and PDB Validation server were used between cycles to monitor the overall quality of the refinements assessing the geometry of the structures and identifying poorly modelled regions.[8],[9]

Supplementary Tables

Supplementary Table 1 | Crystallisation Conditions of $PHD2_{181-407}$-Molidustat and IOX4 Complexes.

Protein Complex	Sample Concentration	Crystallisation Conditions	Vapor Diffusion Conditions
**PHD2-Molidustat**	1 mM PHD2<br>1.2 mM $MnCl_2$<br>2.2 mM succinic acid<br>2.2 mM Molidustat*	0.2 M potassium thiocyanate pH 7<br>21% (w/v) polyethylene glycol 3350	Sitting Drop (300 nL)<br>protein-to-well ratio, 2:1<br>298K
**PHD2-IOX4**	1 mM PHD2<br>1.2 mM $MnCl_2$<br>2.2 mM succinic acid<br>2.2 mM IOX4*	0.15 M potassium thiocyanate pH 7<br>20.5% (w/v) polyethylene glycol 3350	Sitting Drop (300 nL)<br>protein-to-well ratio, 2:1<br>298K

*Inhibitors were dissolved to make 100 mM DMSO stock solutions and incubated with enzyme-succinate complex for 1 h on ice.

Supplementary Table 2 | PHD2$_{181-407}$-Molidustat and IOX4 Complex Data Collection Parameters and Refinement Statistics.

	PHD2 IOX4 Inhibitor Complex (PDB: 6ZBN)	PHD2 Molidustat Inhibitor Complex (PDB: 6ZBO)
**Beamline**	Diamond Light Source-I04	Diamond Light Source-I04
**Detector**	Dectris Eiger2 XE 16M	Dectris Eiger2 XE 16M
**Data Processing**	Xia2 DIALS	Xia2 DIALS
**Wavelength**	0.9795	0.9795
**Resolution range**	19.97 - 2.01 (2.082 - 2.01)	53.71 - 1.79 (1.854 - 1.79)
**Space group**	*P* 1 2$_1$ 1	*P* 1 2$_1$ 1
**Unit cell**	77.0625 75.4718 127.555 90 95.0415 90	77.119 75.1513 127.215 90 95.3071 90
**Total reflections**	194111 (19357)	268399 (26276)
**Unique reflections**	97123 (9699)	134369 (13146)
**Multiplicity**	2.0 (2.0)	2.0 (2.0)
**Completeness (%)**	99.64 (99.49)	98.25 (96.75)
**Mean I/sigma(I)**	13.41 (1.10)	15.12 (1.19)
**Wilson B-factor**	46.55	36.96
**R-merge†**	0.02276 (0.6275)	0.02229 (0.5392)
**R-meas**	0.03219 (0.8874)	0.03152 (0.7625)
**R-pim**	0.02276 (0.6275)	0.02229 (0.5392)
**CC1/2**	1 (0.752)	0.999 (0.779)
**CC***	1 (0.927)	1 (0.936)
**Reflections used in refinement**	96894 (9654)	133987 (13069)
**Reflections used for R-free**	4823 (494)	6820 (682)
**R-work✧**	0.2056 (0.3615)	0.1776 (0.3274)
**R-free✧**	0.2301 (0.3792)	0.2033 (0.3262)
**CC(work)**	0.963 (0.805)	0.963 (0.890)
**CC(free)**	0.956 (0.730)	0.951 (0.881)
**Number of non-hydrogen atoms**	9865	10381
**macromolecules**	9421	9599
**ligands**	156	157
**solvent**	288	625
**Protein residues**	1197	1218
**RMS(bonds)**	0.002	0.006
**RMS(angles)**	0.47	0.75
**Ramachandran favored (%)**	96.93	97.80
**Ramachandran allowed (%)**	3.07	2.20
**Ramachandran outliers (%)**	0	0
**Rotamer outliers (%)**	1.45	1.61
**Clashscore**	2.36	2.78
**Average B-factor**	67.10	52.56
**macromolecules**	67.66	52.94
**ligands**	51.68	42.77
**solvent**	57.11	49.24
**Number of TLS groups**	39	43

Statistics for the highest-resolution shell are shown in parentheses.

† Rmerge (or Rsym) is equal to ∑|I-<I>|/∑I. Rmerge represents the data quality of merged reflection data. *I* is equal to the intensity of individual measurments and <*I*> is equal to the average of multiple measurements.

✧R_{free} is the R_{factor} for 5% of the reflections which are excluded during refinement.

R_{factor} is equal to ∑hkl||Fobs(hkl)| − k |Fcalc(hkl)||/∑hkl|Fobs(hkl)| and calculated for the working set of reflections (R_{work}).

Supplementary Table 3 | Crystallisation Conditions of PHD2$_{181\text{-}407}$-Inhibitor Complexes Deposited into the Protein Data Bank.[1,10–18]

PDB ID	2G1M	3OUI	3OUH	4BQX	4BQY	4JZR	4KBZ	5A3U	5OX5	5OX6	5V18	6NMQ	6QGV	6ST3	6ZBN	6ZBO
**Space Group**	*P* 6_3	*P* 2_1 2_1 2_1	*P* 6_3	*P* 6_3	*P* 6_3	*P* 6_3	*P* 4_1	*P* 3_2 1 2	*P* 6_3	*P* 6_3	*P* 4_1	*H* 3 2	*H* 3 2	*P* 2_1 2 2_1	*P* 2_1	*P* 2_1
**Resolution**	2.20	1.70	2.51	1.79	1.55	2.10	2.15	3.30	2.25	1.99	2.15	1.58	1.40	2.43	2.01	1.70
**MOL/ASU**	1	1	1	1	1	1	1	3	1	1	1	1	1	2	6	6
**Ligand**	4HG	42Z	014	UN9	FNT	4JR	1QA	R8J	B2E	A1Z	8UY	KU1	J2H	LUW	QEE	QEQ
**Salt**	$(NH_4)_2SO_4$	$(NH_4)_2SO_4$	$(NH_4)_2SO_4$	$(NH_4)_2SO_4$	$(NH_4)_2SO_4$	$(NH_4)_2SO_4$	$(NH_4)_2SO_4$	sodium citrate / citric acid	sodium citrate tribasic dehydrate	sodium acetate tribasic dehydrate		$(NH_4)_2SO_4$	$(NH_4)_2SO_4$	sodium citrate tribasic dihydrate	KSCN	KSCN
		NaOAc	$MgSO_4$			NaOAc				$(NH_4)_2SO_4$		$MgSO_4$		NaOAc	succinic acid	succinic acid
**Precipitant**	dioxane	PEG 400		dioxane	dioxane	PEG 3350	PEG 8000			PEG 8000	PEG 3350		2-propanol	PEG 4000	PEG 3350	PEG 3350
**Buffer/Additive**	MES		Imidazole	MES	MES		HEPES				Bis-Tris/ASO4	Imidazole			Tris-HCl	Tris-HCl
**Glycerol (%)**	0	0	10	1	1		0	1	1	1	0	10	1	1	1	1
**pH**	6.5	5.5	6.5	6.5	6.5	4.8	7.2	6.5	6.5	4.6	6.5	6.5	7.5	5.6	7	7

PDB ID	***Reference***	***Ligands***
**2G1M**	**10**	***4HG:*** *N-(4-hydroxy-8-iodoisoquinoline-3-carbonyl)glycine*
**3OUI**	**11**	***42Z:*** *N-[(5,6-dichloro-1H-benzimidazol-2-yl)carbonyl]glycine*
**3OUH**	**11**	***014:*** *1-(5-chloro-6-fluoro-1H-benzimidazol-2-yl)-1H-pyrazole-4-carboxylic acid*
**4BQX**	**12**	***UN9:*** *N-(1-chloro-4-hydroxyisoquinoline-3-carbonyl)glycine*
**4BQY**	**12**	***FNT:*** *(2S)-2-{[(1-chloro-4-hydroxyisoquinolin-3-yl)carbonyl]amino}propanoic acid*
**4JZR**	**13**	***4JR:*** *2-(biphenyl-4-yl)-8-[(1-methyl-1H-imidazol-2-yl)methyl]-2,8-diazaspiro[4.5]decan-1-one*
**4KBZ**	**NA**	***1QA:*** *(2S)-({[2-(5-cyano-3-hydroxypyridin-2-yl)-1,3-thiazol-4-yl]acetyl}amino)(phenyl)ethanoic acid*
**5A3U**	**1**	***R8J:*** *6-(5-oxo-4-(1H-1,2,3-triazol-1-yl)-2,5-dihydro-1H-pyrazol-1-yl)nicotinic acid*
**5OX5**	**15**	***B2E:*** *(6-hydroxy-1,3-dimethyl-2,4-dioxo-1,2,3,4-tetrahydropyrimidine-5-carbonyl)glycine*
**5OX6**	**15**	***AZ1:*** *2-[[5-(3-chlorophenyl)-3-oxidanyl-pyridin-2-yl]carbonylamino]ethanoic acid*
**5V18**	**16**	***8UY:*** *4-([1,2,4]triazolo[1,5-a]pyridin-5-yl)benzonitrile*
**6NMQ**	**17**	***KU1:*** *N-(4-oxo-1,4-dihydrocinnoline-3-carbonyl)glycine*
**6QGV**	**18**	***J2H:*** *8-[(3-methylpyridin-2-yl)methyl]-3-(4-phenylphenyl)-1-pyrimidin-2-yl-1,3,8-triazaspiro[4.5]decane-2,4-dione*
**6ST3**	**19**	***LUW:*** *4-oxidanyl-~{N}-[(4-phenoxyphenyl)methyl]-2-pyrazol-1-yl-pyrimidine-5-carboxamide*
**6ZBN**	**Current**	***QEE:*** *tert-butyl 6-(5-hydroxy-4-(1H-1,2,3-triazol-1-yl)-1H-pyrazol-1-yl)nicotinate*
**6ZBO**	**Current**	***QEQ:*** *1-(6-morpholinopyrimidin-4-yl)-4-(1H-1,2,3-triazol-1-yl)-1H-pyrazol-5-ol*

Supplementary Table 4 | RMSD (Å) Comparison of the Conformations of Chains A-F in the PHD2$_{181-407}$-Molidustat and IOX4 Inhibitor Complex Structures.

Chain	Molidustat (PDB ID: 6ZBO)	IOX4 (PDB ID: 6ZBN)
A	***	0.169
B	0.262	0.307
C	0.199	0.207
D	0.211	0.225
E	0.169	0.253
F	0.285	0.351

*Structure Comparison Tool - PHENIX.

*** Reference model chain for RMSD comparisons.

Supplementary Figures

Supplementary Figure 1 | Overview of PHD2 Inhibitor Structures and IUPAC Names. Structures and IUPAC names of PHD inhibitors, Molidustat (Bayer Pharmaceuticals, Germany), IOX4 (Bayer Pharmaceuticals, Germany), IOX4-acid (Oxford, UK), Takeda-17 (Takeda Pharmaceuticals), Roxadustat (FibroGen), Vadadustat (Akebia Therapeutics), FG-2216 (FibroGen) and JPHM-2-167 (Oxford) referenced in this work.[1,14,15,17,19]

Molidustat

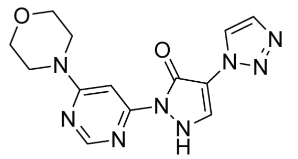

2-(6-morpholinopyrimidin-4-yl)-4-(1*H*-1,2,3-triazol-1-yl)-1,2-dihydro-3*H*-pyrazol-3-one

IOX4

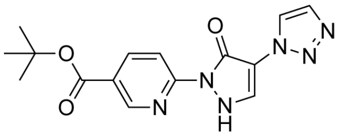

tert-butyl 6-(5-oxo-4-(1*H*-1,2,3-triazol-1-yl)-2,5-dihydro-1*H*-pyrazol-1-yl)nicotinate

IOX4-A

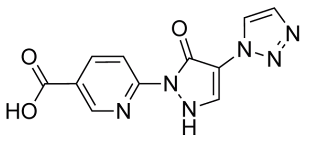

6-(5-oxo-4-(1*H*-1,2,3-triazol-1-yl)-2,5-dihydro-1*H*-pyrazol-1-yl)nicotinic acid

Takeda-17

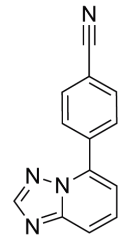

4-([1,2,4]triazolo[1,5-a]pyridin-5-yl)benzonitrile

Roxadustat

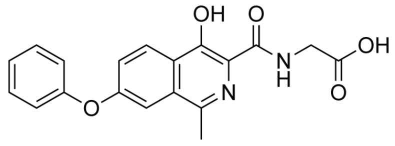

(4-hydroxy-1-methyl-7-phenoxy isoquinoline-3-carbonyl)glycine

Vadadustat

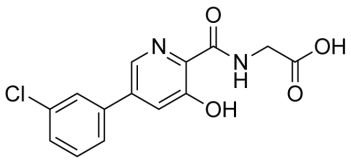

N-{[5-(3-chlorophenyl)-3-hydroxy-2-pyridinyl]carbonyl}glycine

FG-2216

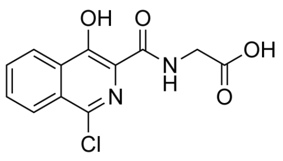

N-{[5-(3-chlorophenyl)-3-hydroxy-2-pyridinyl]carbonyl}glycine

Daprodustat

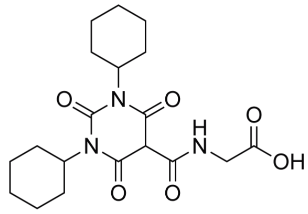

(1,3-dicyclohexyl-2,4,6-trioxohexahydropyrimidine-5-carbonyl)glycine

JPHM-2-167

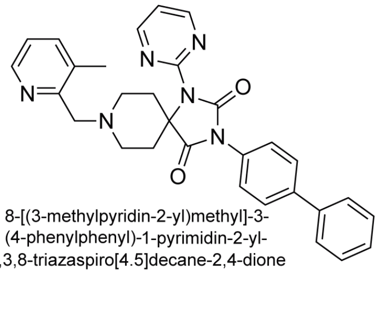

8-[(3-methylpyridin-2-yl)methyl]-3-(4-phenylphenyl)-1-pyrimidin-2-yl-1,3,8-triazaspiro[4.5]decane-2,4-dione

Supplementary Figure 2 | *P*2$_1$ Crystallisation System Lattice Formation of Asymmetric Unit (PDB: 6ZBO). The protein surface of the asymmetric unit of the PHD2$_{181\text{-}407}$-Molidustat complex structure is depicted below at a resolution of 1.79Å. Roman numerals refer to the eight β-strand (I-VIII) of the core DSBH fold of the 2OG oxygenases. Protomers are colour coordinated based on each dimer pair α4-helix interactions.[2,3]

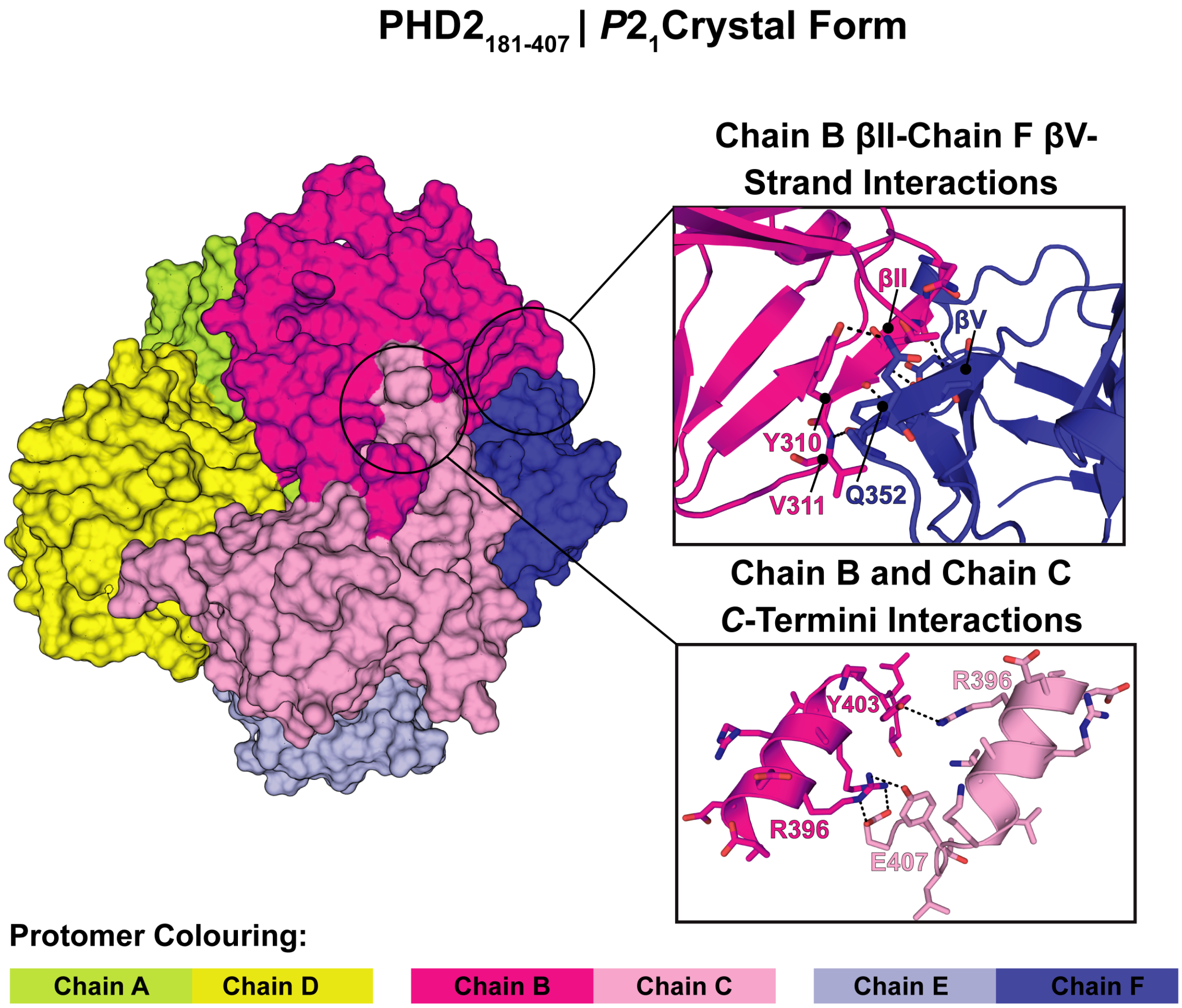

Supplementary Figure 3 | Comparison of FIH and PHD2 Active Sites Complexed with Molidustat in Complex. (PDB: 5OP8 and 6ZBO). (A) View of the active site of FIH in complex with Molidustat (PDB: 5OP8). Molidustat binds in a similar, but not identical, mode to FIH as it does to PHD2. The triazole ring of Molidustat interacts with Lys214 and Tyr145 of FIH that bind the C5 carboxylate of 2OG during catalysis. (B and D) 2D active site interactions are shown in red dashes with corresponding distance in Å. (B) 2D active site interactions of FIH in complex with Molidustat. (C-D) 2D active site interactions and view of the PHD2-Molidustat inhibitor complex (PDB: 6ZBO).

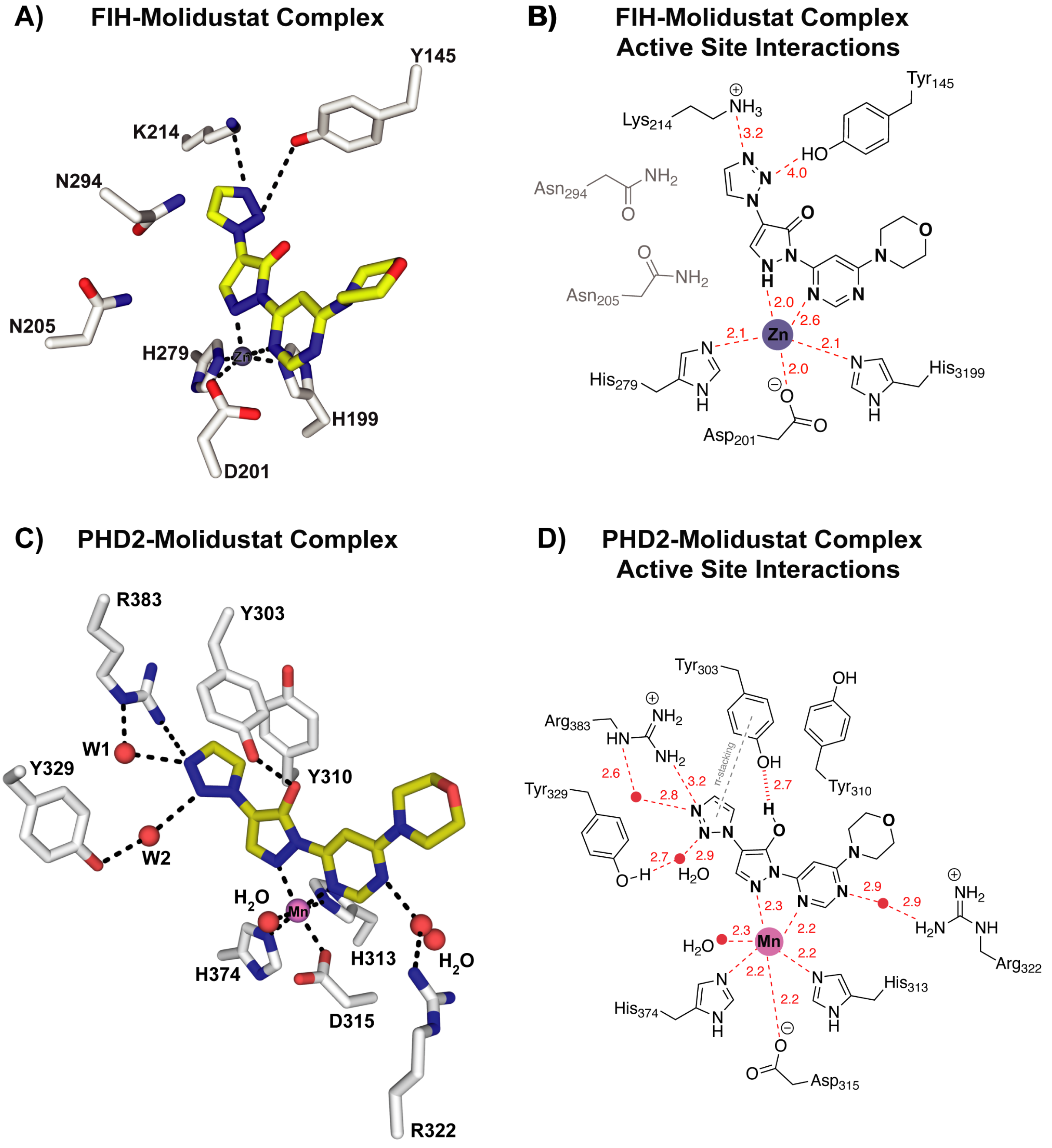

Supplementary Figure 4 | 2D Active Site Interactions of PHD2 in Complex with Vadadustat, FG-2216, Takeda-17, or IOX4-A (PDB: 5OX6, 4BQX, 5V18, 5A3U). Active site interactions are shown with red dashes corresponding to distances in Å.

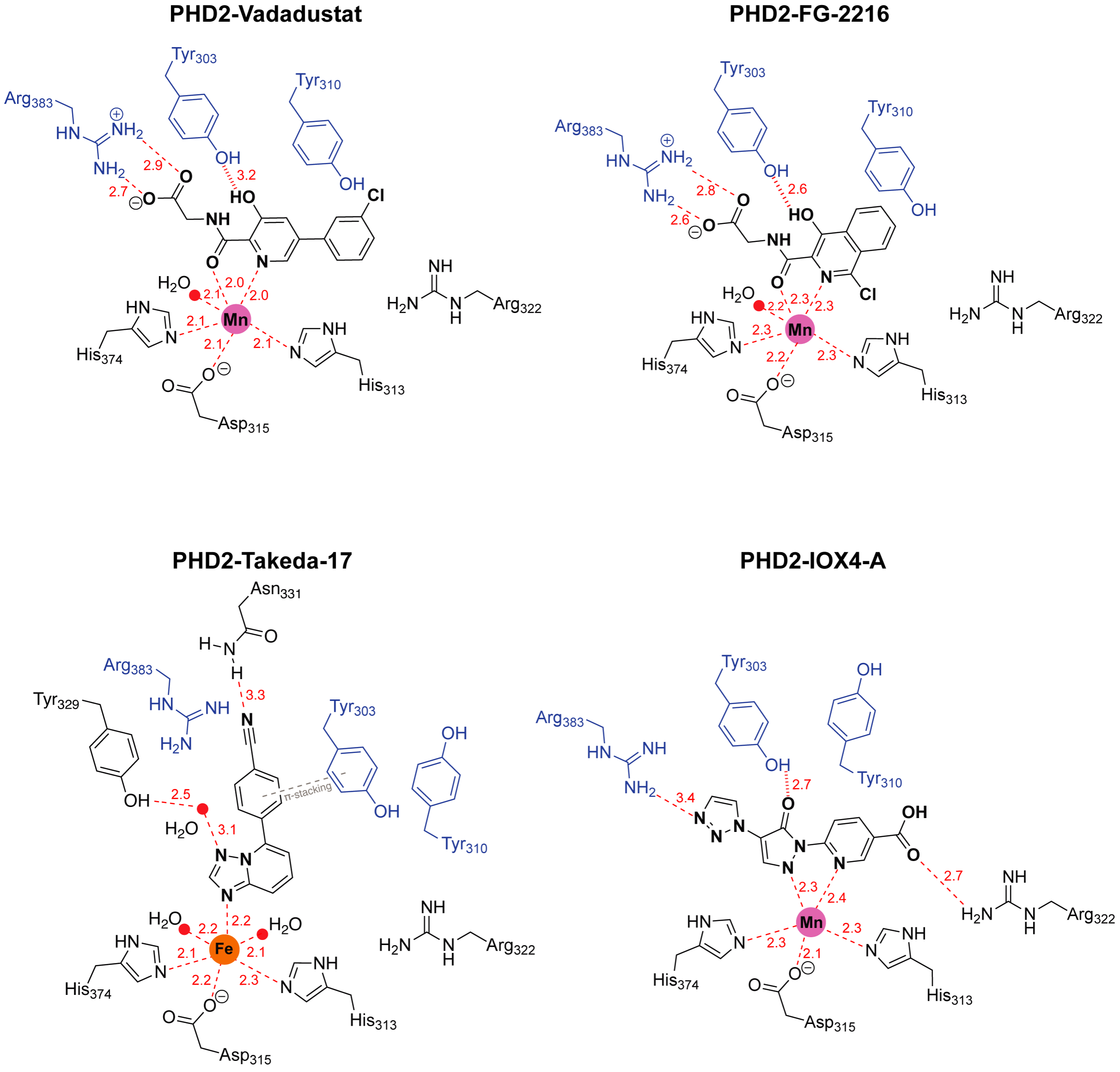

References

[1] M. C. Chan, O. Atasoylu, E. Hodson, A. Tumber, I. K. H. Leung, R. Chowdhury, V. Gómez-Pérez, M. Demetriades, A. M. Rydzik, J. Holt-Martyn, Y.-M. Tian, T. Bishop, T. D. W. Claridge, A. Kawamura, C. W. Pugh, P. J. Ratcliffe, C. J. Schofield, *PLoS One* **2015**, *10*, e0132004.

[2] R. Chowdhury, I. K. H. H. Leung, Y.-M. M. Tian, M. I. Abboud, W. Ge, C. Domene, F.-X. F. X. Cantrelle, I. Landrieu, A. P. Hardy, C. W. Pugh, P. J. Ratcliffe, T. D. W. W. Claridge, C. J. Schofield, *Nat. Commun.* **2016**, *7*, 12673.

[3] R. Chowdhury, M. A. McDonough, J. Mecinović, C. Loenarz, E. Flashman, K. S. Hewitson, C. Domene, C. J. Schofield, *Structure* **2009**, *17*, 981–989.

[4] G. Winter, C. M. C. Lobley, S. M. Prince, *Acta Crystallogr. Sect. D Biol. Crystallogr.* **2013**, *69*, 1260–1273.

[5] A. J. McCoy, R. W. Grosse-Kunstleve, P. D. Adams, M. D. Winn, L. C. Storoni, R. J. Read, *J. Appl. Crystallogr.* **2007**, *40*, 658–674.

[6] P. Emsley, K. Cowtan, *Acta Crystallogr. Sect. D Biol. Crystallogr.* **2004**, *60*, 2126–2132.

[7] P. D. Adams, P. V. Afonine, G. Bunkóczi, V. B. Chen, I. W. Davis, N. Echols, J. J. Headd, L. W. Hung, G. J. Kapral, R. W. Grosse-Kunstleve, A. J. McCoy, N. W. Moriarty, R. Oeffner, R. J. Read, D. C. Richardson, J. S. Richardson, T. C. Terwilliger, P. H. Zwart, *Acta Crystallogr. Sect. D Biol. Crystallogr.* **2010**, *66*, 213–221.

[8] V. B. Chen, W. B. Arendall, J. J. Headd, D. A. Keedy, R. M. Immormino, G. J. Kapral, L. W. Murray, J. S. Richardson, D. C. Richardson, *Acta Crystallogr. Sect. D Biol. Crystallogr.* **2010**, *66*, 12–21.

[9] S. Gore, E. Sanz García, P. M. S. Hendrickx, A. Gutmanas, J. D. Westbrook, H. Yang, Z. Feng, K. Baskaran, J. M. Berrisford, B. P. Hudson, Y. Ikegawa, N. Kobayashi, C. L. Lawson, S. Mading, L. Mak, A. Mukhopadhyay, T. J. Oldfield, A. Patwardhan, E. Peisach, G. Sahni, M. R. Sekharan, S. Sen, C. Shao, O. S. Smart, E. L. Ulrich, R. Yamashita, M. Quesada, J. Y. Young, H. Nakamura, J. L. Markley, H. M. Berman, S. K. Burley, S. Velankar, G. J. Kleywegt, *Structure* **2017**, *25*, 1916–1927.

[10] M. A. McDonough, V. Li, E. Flashman, R. Chowdhury, C. Mohr, B. M. R. Lienard, J. Zondlo, N. J. Oldham, I. J. Clifton, J. Lewis, L. A. McNeill, R. J. M. Kurzeja, K. S. Hewitson, E. Yang, S. Jordan, R. S. Syed, C. J. Schofield, *Proc. Natl. Acad. Sci.* **2006**, *103*, 9814–9819.

[11] M. D. Rosen, H. Venkatesan, H. M. Peltier, S. D. Bembenek, K. C. Kanelakis, L. X. Zhao, B. E. Leonard, F. M. Hocutt, X. Wu, H. L. Palomino, T. I. Brondstetter, P. V. Haugh, L. Cagnon, W. Yan, L. A. Liotta, A. Young, T. Mirzadegan, N. P. Shankley, T. D. Barrett, M. H. Rabinowitz, *ACS Med. Chem. Lett.* **2010**, *1*, 526–529.

[12] R. Chowdhury, J. I. Candela-Lena, M. C. Chan, D. J. Greenald, K. K. Yeoh, Y.-M. Tian, M. A. McDonough, A. Tumber, N. R. Rose, A. Conejo-Garcia, M. Demetriades, S. Mathavan, A. Kawamura, M. K. Lee, F. van Eeden, C. W. Pugh, P. J. Ratcliffe, C. J. Schofield, *ACS Chem. Biol.* **2013**, *8*, 1488–1496.

[13] G. Deng, B. Zhao, Y. Ma, Q. Xu, H. Wang, L. Yang, Q. Zhang, T. B. Guo, W. Zhang, Y. Jiao, X. Cai, J. Zhang, H. Liu, X. Guan, H. Lu, J. Xiang, J. D. Elliott, X. Lin, F. Ren, *Bioorg. Med. Chem.* **2013**, *21*, 6349–6358.

[14] T.-L. Yeh, T. M. Leissing, M. I. Abboud, C. C. Thinnes, O. Atasoylu, J. P. Holt-Martyn, D. Zhang, A. Tumber, K. Lippl, C. T. Lohans, I. K. H. Leung, H. Morcrette, I. J. Clifton, T. D. W. Claridge, A. Kawamura, E. Flashman, X. Lu, P. J. Ratcliffe, R. Chowdhury, C. W. Pugh, C. J. Schofield, *Chem. Sci.* **2017**, *8*, 7651–7668.

[15] S. Ahmed, A. Ayscough, G. R. Barker, H. E. Canning, R. Davenport, R. Downham, D.

Harrison, K. Jenkins, N. Kinsella, D. G. Livermore, S. Wright, A. D. Ivetac, R. Skene, S. J. Wilkens, N. A. Webster, A. G. Hendrick, *J. Med. Chem.* **2017**, *60*, 5663–5672.

[16] S. D. Bembenek, H. Venkatesan, H. M. Peltier, M. D. Rosen, T. D. Barrett, K. C. Kanelakis, H. L. Palomino, T. I. Brondstetter, T. Mirzadegan, M. H. Rabinowitz, *ACS Omega* **2019**, *4*, 6703–6708.

[17] J. P. Holt-Martyn, A. Tumber, M. Z. Rahman, K. Lippl, W. Figg, M. A. McDonough, R. Chowdhury, C. J. Schofield, *Med.Chem.Comm.* **2019**, *10*, 500–504.

[18] J. P. Holt-Martyn, R. Chowdhury, A. Tumber, T. Yeh, M. I. Abboud, K. Lippl, C. T. Lohans, G. W. Langley, W. Figg, M. A. McDonough, C. W. Pugh, P. J. Ratcliffe, C. J. Schofield, *ChemMedChem* **2020**, *15*, 270–273.

[19] H. Beck, M. Jeske, K. Thede, F. Stoll, I. Flamme, M. Akbaba, J.-K. Ergüden, G. Karig, J. Keldenich, F. Oehme, H.-C. Militzer, I. V Hartung, U. Thuss, *ChemMedChem* **2018**, *13*, 988–1003.
